# Lack of cortisol response in patients with posttraumatic stress disorder (PTSD) undergoing a diagnostic interview

**DOI:** 10.1186/1471-244X-7-54

**Published:** 2007-10-04

**Authors:** Iris-Tatjana Kolassa, Cindy Eckart, Martina Ruf, Frank Neuner, Dominique JF de Quervain, Thomas Elbert

**Affiliations:** 1 Clinical & Neuropsychology, University of Konstanz, Universitätsstr. 10, 78457 Konstanz, Germany; 2 Division of Psychiatry Research, University of Zürich, Lenggstr. 31, 8032 Zürich, Switzerland

## Abstract

**Background:**

According to DSM-IV, the diagnosis of posttraumatic stress disorder (PTSD) requires the experience of a traumatic event during which the person's response involved intense fear, helplessness, or horror. In order to diagnose PTSD, clinicians must interview the person in depth about his/her previous experiences and determine whether the individual has been traumatized by a specific event or events. However, asking questions about traumatic experiences can be stressful for the traumatized individual and it has been cautioned that subsequent "re-traumatization" could occur. This study investigated the cortisol response in traumatized refugees with PTSD during a detailed and standardized interview about their personal war and torture experiences.

**Methods:**

Participants were male refugees with severe PTSD who solicited an expert opinion in the Psychological Research Clinic for Refugees of the University of Konstanz. 17 patients were administered the *Vivo *Checklist of War, Detention, and Torture Events, a standardized interview about traumatic experiences, and 16 subjects were interviewed about absorption behavior. Self-reported measures of affect and arousal, as well as saliva cortisol were collected at four points. Before and after the experimental intervention, subjects performed a Delayed Matching-to-Sample (DMS) task for distraction. They also rated the severity of selected PTSD symptoms, as well as the level of intrusiveness of traumatic memories at that time.

**Results:**

Cortisol excretion diminished in the course of the interview and showed the same pattern for both groups. No specific response was detectable after the supposed stressor. Correspondingly, ratings of subjective well-being, memories of the most traumatic event(s) and PTSD symptoms did not show any significant difference between groups. Those in the presumed stress condition did not perform worse than persons in the control condition after the stressor. However, both groups performed poorly in the DMS task, which is consistent with memory and concentration problems demonstrated in patients with PTSD.

**Conclusion:**

A comprehensive diagnostic interview including questions about traumatic events does not trigger an HPA-axis based alarm response or changes in psychological measures, even for persons with severe PTSD, such as survivors of torture. Thus, addressing traumatic experiences within a safe and empathic environment appears to impose no unacceptable additional load to the patient.

## Background

Severe traumatic experiences such as torture and war frequently produce long-term psychological effects that can persist over decades, and even into old age [1]. During torture, the individual is rendered completely helpless and is often overwhelmed by fear and horror, which is likely to cause a trauma-related psychological disorder. Torture victims frequently experience symptoms of PTSD, as delineated by the DSM-IV [2]. These symptoms include: recurrent memories of the traumatic event in the form of intrusions and nightmares, avoidance of thoughts and/or places associated with the traumatic event, enhanced vigilance and hyperarousal, sleep disturbances, and emotional numbing. If these symptoms persist for more than one month, posttraumatic stress disorder (PTSD) is diagnosed. Prevalence rates of PTSD between 45% and over 90% have been reported in survivors of torture [1,3,4].

There is growing evidence that extremely stressful adverse experiences have a lasting impact on the neurobiology of the stress response, involving both baseline (*tonic*) abnormalities [5,6] and abnormal responsivity (*phasic *abnormalities) of the hormonal stress system [5,7]. Stress leads to the excretion of corticotrophin-releasing factor (CRF) and arginine vasopressin (AVP) into the hypophysial blood supply, where they are transported to the adenohypophysis. Here they activate pituitary corticoptrophs to synthesize and release adrenocorticotrophic hormone (ACTH) into the general blood circulation. CRH is the most potent ACTH secretagogue. Although the role of AVP is not yet completely understood, it seems to be involved in the regulation of stress-induced ACTH release [8]. ACTH circulates in the blood to the zona fasciculata of the adrenal cortex, where it promotes the conversion of cholesterol esters into free cholesterol and ultimately results in the release of cortisol as the end product of the steroid pathway from the adrenal cortex into the circulatory system. Cortisol peaks are typically seen 15–30 minutes after an ACTH pulse in normal human subjects [for a summary see [9]]. After an acute psychological stressor, peak cortisol responses occur 20–40 minutes from the onset of the stressor in healthy controls, with the length of the stressor not associated with observed effect sizes (i.e. longer stressors do not lead to greater cortisol responses than shorter ones [10]).

Several studies have investigated the effect of traumatic life experiences and PTSD on the pituitary-adrenal cortical system. However, results have been inconsistent and sometimes even contradictory [for an overview see e.g., [5,7]]. According to the original *glucocorticoid cascade hypothesis *[11], chronic exposure to glucocorticoids throughout life, secondary to repeated or traumatic stress, downregulates the central glucocorticoid receptors, especially at the hippocampal level. This causes impairment of HPA sensitivity to the negative steroid feedback, leading to glucocorticoid overproduction in a feedforward loop. In contrast to this original hypothesis, several studies reported lower baseline cortisol levels in PTSD in plasma [12-14], saliva [15-17], and urine [18-21], but some studies have found significantly higher cortisol excretion [e.g., [22]] or no differences between groups [e.g., [23-25]].

In addition to the investigation of baseline alterations in PTSD patients, several challenge paradigms have been developed to investigate the stress response in PTSD patients. These paradigms can be broadly distinguished in pharmacological and non-pharmacological challenge tests [for a review see [7]]. Pharmacological challenge tests target the HPA axis at different levels, e.g. dexamethasone suppression test, ACTH-, CRH-, and naloxone challenges, dexamethasone CRH test and metyrapone challenge designs. In their review, de Kloet et al. [7] conclude that enhanced cortisol suppression after administration of .5 mg dexamethasone is a relatively well-corroborated finding. Otherwise the results of pharmacological challenge paradigms are still inconclusive.

In comparison to pharmacological challenge tests relatively few non-pharmacological challenge tests have been conducted with PTSD patients [26-30]. In these paradigms, cortisol levels were investigated after cognitive [29], psychosocial [30], or physical challenges [28], as well as in response to personalized traumatic scripts [27] and trauma reminders [26].

Challenge paradigms using non-trauma-related stressors such as psychosocial or physical stressors found mixed results: Using a cognitive challenge task, Bremner et al. [29] found higher mean salivary cortisol levels in anticipation of, and during the stressor in PTSD patients compared to controls. This effect was more pronounced in male PTSD patients compared with female PTSD patients. However, aside from these baseline differences, no evidence for a changed cortisol response to cognitive stressors in PTSD was observed. Instead, the cortisol response was similar in the PTSD and control group. Using a physical stressor (cold pressor task), Santa Ana et al. [28] investigated ACTH/cortisol excretion in individuals with PTSD, comorbid alcohol dependence and PTSD, and controls. In this paradigm, subjects immerse one hand in a cold water bath for up to 1 minute or as long as they can tolerate. Regardless of the presence or absence of comorbid alcohol dependence, subjects with childhood trauma and PTSD (but not adult trauma and PTSD) had lower plasma cortisol at baseline and at all post-task measurement points. No differences in cortisol reagibility were observed. However, while control persons exhibited an initial ACTH increase in response to the stressor, traumatized persons (childhood and adult trauma) showed a blunted ACTH excretion. In contrast, Heim et al. [30] reported a persistent sensitization of the HPA axis in adulthood even to mild stressors in women with severe early-life stress. In response to a psychosocial laboratory stressor, physically and sexually abused women with and without current major depression exhibited increased ACTH concentrations compared to controls and non-abused depressed women. In contrast, only women with early-life stress and current major depression showed enhanced plasma cortisol values 30 to 60 minutes after the stressor.

The results of challenge paradigms using trauma-related stressors, such as trauma reminders or personalized traumatic scripts are also inconclusive. In a symptom provocation study using trauma-related stimuli (combat sounds) compared to nonspecific arousing stimuli (white noise), Liberzon et al. [26] observed enhanced plasma cortisol and catecholamines at baseline, but no differences in ACTH or cortisol secretion in response to both stressors (combat sounds, white noise) in patients with PTSD compared to controls. One major problem with this study is that plasma samples were drawn immediately before and after playing a 3 minute audiotape. An effect of a stressor on cortisol values cannot be expected after such a short time interval. On the other hand, Elzinga et al. [27] reported elevated salivary cortisol levels in women with PTSD during, and shortly after, exposure to a personalized trauma script. The problem in this study is that the cortisol levels were elevated before the script exposure in the PTSD group compared to the control group, perhaps due to anticipatory anxiety. It is unclear whether effects would have been significant if the difference values had been analyzed or if the baseline differences had been corrected using cortisol values directly before the stressor as a covariate.

Finally, in a single case study Otte et al. [31] investigated subjective distress and salivary cortisol during the first and 20^th ^session of an imaginal exposure treatment. They observed extreme anticipatory anxiety before and in the beginning of the first exposure session, which was markedly reduced at the end of the therapy. However, they observed no increase in cortisol during first exposure, and cortisol values at the end of the therapy were not different from the beginning.

In summary, the results of non-pharmacological challenge paradigms are as inconclusive as the results of pharmacological challenge paradigms in PTSD. Studies using cognitive, psychosocial, or physical stressors revealed no evidence of abnormalities in cortisol reagibility [28-30]. However, it remains unclear whether findings from cognitive, psychosocial, or physical challenge paradigms can be generalized to emotional stressors, such as being exposed to reminders of one's trauma. To our knowledge, only two studies so far investigated cortisol secretion in PTSD patients during or after exposure to trauma-related stimuli with conflicting results: One found no increase in plasma cortisol in response to the stressor [26], whereas the other found increased cortisol to personalized traumatic scripts [27]. However, as detailed above, both studies had limitations that make the interpretation of results difficult.

Thus, it remains unclear whether inquiring about a traumatic experience, for example during a psychodiagnostic interview, for expert opinion, or in the course of exposure-based therapies, such as narrative exposure therapy [32], causes a level of stress that activates the HPA axis and results in excessive cortisol excretion in PTSD patients. Therefore, the aim of this study was to investigate whether persons with severe forms of PTSD, such as survivors of torture and violent oppression, show an excessive stress response with high cortisol excretion in response to detailed questioning about their torture/war experiences. Samples of saliva were taken four times during a diagnostic interview (baseline, pre-stressor, post-stressor, recovery). Individuals with PTSD in the stress group were interviewed about their torture experiences using a standardized checklist. Persons with PTSD in the control condition were interviewed about their absorption behavior in various situations. Directly before and after the stressor, all participants performed a memory task, which acted as a distraction, and rated selected PTSD symptoms on a visual analog scale.

We hypothesized that participants in the stress group would show an excessive cortisol excretion after being questioned about their traumatic (torture/war) experiences. Furthermore, we wanted to investigate differences in recovery of cortisol levels between the two groups after the stressor. In addition, we hypothesized that after the interview persons in the stress group would show worse performance on the memory task and would rate their PTSD symptoms and memories as more severe than persons in the control group. Finally, we wanted to explore the relationship between cortisol levels and severity of symptom ratings and memories of the most traumatic event pre- and poststressor.

## Methods

### Participants

Participants were male refugees suspected by social workers of having PTSD. They were invited to the Psychological Research Clinic for Refugees of the University of Konstanz, located at the Center for Psychiatry, Reichenau. Travel expenses and interpreter costs were paid for the participants. If resources permitted, they were offered treatment; otherwise they were referred to local therapists. In the case of ongoing court procedures regarding the state of asylum, they were offered a brief report on their mental health status. The procedure was approved by the Ethics Committee of the University of Konstanz, Germany.

Forty-four male refugees participated in the study. Eleven participants were excluded from the study: 6 participants did not fulfill DSM-IV criteria of PTSD, in 2 cases the diagnosis of PTSD was doubtful, 1 participant suffered from an anxiety attack with vomiting at the time of the investigation, 1 participant had undergone thyroid gland operation and took hormone substituting medication, and 1 refused to give a sample of saliva. The remaining 33 participants were assigned at random to the stress condition (*n *= 17) and to the control condition (*n *= 16). The included refugees had the following ethnic backgrounds: 26 Kurds from Turkey, 2 Kosovar Albanians, 2 Afghans, 1 Kosovar Roma, 1 Syrian, and 1 Moroccan. Refugees' mean age was 34 years (*SD *= 7.6; age range 22–50 years). There was no significant age difference between groups, *t*(31)= -.82, *p *= .42 (*M*_*Controls *_= 33, *SD *= 7.9; *M*_*stress group *_= 35, *SD *= 7.4). On average participants lived in Germany for 5.3 years (*SD *= 3.2). Eight persons of the stress group and 8 persons of the control group were smokers.

The sample can be considered as severely traumatized: On average each participant had experienced 5.4 traumatic events (*SD *= 1.6) as measured by the PDS event scale, including in almost all cases torture (n = 30; 16 in the experimental group, 14 in the control group). Nine participants took antidepressants (stress group: 4, control group: 5) and 6 neuroleptics (stress group: 3, control group: 3). Two participants fulfilled criteria of substance dependence, three abused alcohol as a means of self-medication. Thirty-three participants were suicidal (15 mild, 9 moderate, 9 severe). Sixteen participants were smokers (*M *= 13.3 cigarettes/day, *SD *= 15.8).

### Experimental procedure

**Figure 1:**
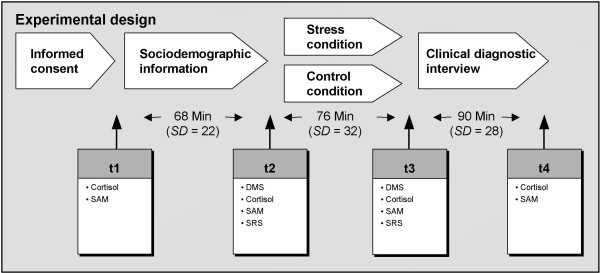
**Experimental design**. SAM, Self Assessment Manikin; SRS, Symptom Rating Scale; DMS, Delayed matching-to-sample task. After obtaining informed consent subjects gave the first saliva probe and rated their current emotional state via SAM (t_1_). The diagnostic interview continued with gathering of sociodemographic information. Before the groups were split in a stress and a control group, a memory test (DMS) was performed for purpose of distraction. Afterwards a sample of saliva was taken, and participants completed the SAM followed by the SRS (t_2_). Subsequently, participants in the stress condition were interviewed about their traumatic (torture) experiences, whereas participants in the control condition were asked about their absorption behavior. Afterwards, subjects again completed the DMS for the purpose of distraction. Then they gave a sample of saliva, and subsequently completed the SAM followed by the SRS (t_3_). After that, information about comorbid psychiatric disorders was gathered in a clinical diagnostic interview. At the end of the interview, participants gave the fourth saliva sample and completed the SAM (t_4_).

Diagnostic interviews started at 10 am and lasted on average about 5 hours. They were conducted with the help of trained interpreters. For a schematic description of the procedures see Figure 1.

At the beginning, all procedures were explained to participants and written informed consent was obtained. Afterwards, participants gave the first sample of saliva via Salivette sampling devices (Sarstedt, Nürnbrecht, Germany) and rated their present condition via the valence and arousal scale of the Self Assessment Manikin [SAM, [33,34]]. Subsequently, sociodemographic information as well as medical information was acquired and participants were interviewed about their current asylum situation.

In the second part of the interview, participants completed a computerized nonverbal Delayed-Matching-to-Sample test (DMS), in which subjects had to memorize and match complex graphical patterns. The purpose of this test was to distract participants from the content of the first part of the diagnostic interview. Subjects were first presented with a familiarization stimulus for 5 seconds. Then there was a delay period of 5 seconds during which subjects were shown a fixation cross in the middle of the screen. After the delay, two test stimuli were presented side by side for 5 seconds. One of these stimuli was the familiarization stimulus, the other one was a new stimulus, similar to the familiarization stimulus, but different in geometry and color. The subjects' task was to press the button that corresponded to the location of the familiarization stimulus. The next trial started after a 3 second-inter-trial delay. In total, 16 trials were conducted, and the DMS task took about five minutes.

After completing the DMS task, participants rated a second time how pleasant/unpleasant and how calm/aroused they felt at the time and gave a second saliva sample. Subsequently, using a visual analog scale, they rated how stressful they perceived the following major symptoms of PTSD to have been during the previous 4 weeks: nightmares, intrusions, irritability, and disturbed sleep. Furthermore, they rated how intense their memories of the worst traumatic experience were at the time and how stressful and unpleasant the memories of their most traumatic event were at that time. Afterwards, subjects had a 15 minute break.

Afterwards, the procedures for the stress and the control group diverged. In the stress condition, participants were interviewed about their experiences during torture using the *Vivo *Checklist of War, Detention, and Torture events (Note: The German and English version of this questionnaire can be downloaded from the vivo foundation web page [35], see vivo publications). In the non-stress condition participants were interviewed about absorption behavior (e.g., "When something absorbs my mind, I have to make an effort to notice what happens around me"). This phase of the interview took on average 78 minutes (*SD *= 27 minutes). The remainder of the experiment was again identical for stress and control groups.

Participants completed the DMS test a second time for distraction, gave a third saliva sample and completed the SAM rating scale again. Afterwards, they once again rated the intensity of the major symptoms of PTSD during the last 4 weeks, how much they were ruminating about their traumatic experience at that time, and how stressful and unpleasant those memories were at the time. In addition, they were asked whether the memories of their most traumatic experience were more intense, less intense, or unchanged.

In the third phase of the interview, participants were asked about symptoms of posttraumatic stress disorder with the Posttraumatic Diagnostic Scale [PDS, [36]], symptoms of depression and anxiety via the Hopkins Symptom Checklist-25 [HSCL-25, [37]], and current suicidality using part C of the German version [38] of the Mini International Neuropsychiatric Interview [M.I.N.I., [39,40]]. Furthermore, participants were asked about quality and duration of sleep during the last night and during the last 4 weeks with a modified version of the Pittsburg Sleep Quality Index [PSQI, [41]]. For mean questionnaire values and standard errors see Table 1. Approximately one hour into this third phase, subjects once again rated via the SAM their current affective state and arousal and gave a fourth saliva sample. After giving this saliva sample, subjects were able to take as much time as they wanted for a break, following which the remaining questionnaires of this phase were completed.

**Table 1:** Questionnaire values

	Total average		Stress group		Control group		
		
Questionnaire	*M*	*SE*	*M*	*SE*	*M*	*SE*	One-way ANOVA
PDS-Intrusion	10.36	.51	10.12	.68	10.63	.79	n.s.
PDS-Avoidance	12.94	.49	12.88	.71	13.00	.71	n.s.
PDS-Hyperarousal	10.36	.49	9.88	.72	10.88	.65	n.s.
PDS Total	33.67	1.17	32.88	1.53	34.5	1.81	n.s.
HSCL-Anxiety	2.71	.09	2.74	.12	2.68	.14	n.s.
HSCL-Depression	2.80	.09	2.71	.09	2.89	.15	n.s.
h sleep last night	3.18	.31	3.04	.43	3.33	.44	n.s.
Average h sleep last 4 weeks	4.59	.26	4.39	.36	4.78	.37	n.s.

Mean questionnaire values (M) and standard errors (SE) for each group. PDS, Posttraumatic Diagnostic Scale; HSCL, Hopkins Symptom Checklist.

### Analysis of saliva samples

Directly after the diagnostic interview, saliva samples were refrigerated at -18°C. They were sent to an external laboratory for analysis (Prof. Dr. C. Kirschbaum, TU Dresden, Germany), where free cortisol levels in saliva were measured using a commercially available chemiluminescence assay (IBL, Hamburg, Germany). Nine saliva devices did not contain enough saliva for analysis and were coded as "missing" in the analysis (2 at t_1_, 1 at t_2_, 3 at t_3_, 3 at t_4_).

Factors that may influence cortisol levels such as age, medications, alcohol and nicotine consumption, aspects of sleeping behavior and comorbid psychiatric disorders (depression, substance abuse) were recorded. Subjects were not allowed to drink coffee or tea or to smoke during the diagnostic interview.

### Statistical analysis

Baseline valence and arousal ratings (at times t_1 _and t_2_) were analyzed by analyses of variance (ANOVAs) with repeated measures factor Time (t_1_, t_2_) and between factor Group (stress group, control group). Valence and arousal ratings after the stress intervention in the experimental group were analyzed by ANCOVAs with repeated measures factor Time (t_3_, t_4_), between factor Group, and valence and arousal ratings at t_2 _as covariates, respectively.

Visual analog symptom rating scale data as well as reaction times and performance in the DMS task were analyzed by an ANOVA with repeated measures factor Time (t_2_, t_3_) and between factor Group (stress group, control group).

Cortisol data were analyzed using an ANOVA with Group as fixed effect and Time as repeated measure (t_1_, t_2_, t_3_, t_4_). Participants' HSCL-D, PDS-Intrusion, PDS-Avoidance, and PDS-Hyperarousal scores were included as covariates but were excluded if they showed no significant influence. Baseline Cortisol (t_1_, t_2_) was investigated using repeated measures factor Time, cortisol reagibility to the stress intervention (t_2_, t_3_) was investigated by subtracting cortisol levels at t_2 _from levels at t_3 _and analyzing the difference as the dependent variable, and the post-stressor dynamics (t_3_, t_4_) were investigated by subtracting cortisol levels at t_3 _from levels at t_4 _and analyzing the difference as the dependent variable [42]. One subject was excluded from the analysis of cortisol data at t_3 _and t_4 _because he became highly aggressive during the interview and appeared as an extreme outlier in cortisol levels.

Data were analyzed using SPSS 13.0. All analyses used mixed model AN(C)OVA, which is particularly suited for analysis of data with missing values [43]. Subject was nested in Group and included as a random effect in all analyses.

## Results

### Analysis of cortisol data

**Figure 2:**
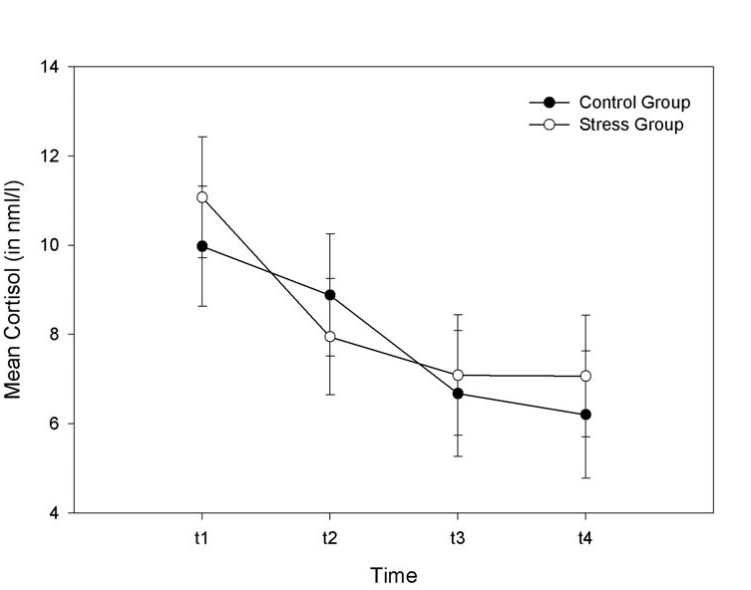
**Cortisol results**. Time course of mean cortisol levels (LSM and SE) for each group.

The ANOVA revealed a main effect of Time, *F*(3,75.38) = 4.31, *p *= .007, showing that cortisol values decreased in both groups over time with no significant difference between groups, interaction Time × Group, *F*(3,75.38) = .63, *p *= .60 (compare Figure 2).

The analysis of baseline cortisol (t_1 _and t_2_) revealed no significant differences between groups, *F*(1,31.02) = .002. However, there was a trend for a main effect of Time, *F*(1,29.46) = 3.88, *p *= .06, indicating lower cortisol values at t_2 _compared to t_1_. Covariates revealed no significant influence and were therefore not included in the ANOVA.

The analysis of cortisol reagibility in response to the stressor (cortisol level at t_2 _subtracted from cortisol level at t_3_) revealed also no effect of Group, *F*(1,27) = 1.79, *p *= .19. Again, covariates revealed no significant influence and were therefore not included in the ANOVA. The correlations of differences in cortisol (t_3_-t_2_) with differences in scores (t_3_-t_2_) in visual analog symptom rating scales also revealed no significant effects.

Furthermore, the analysis of cortisol dynamics after the stressor (cortisol level at t_3 _subtracted from cortisol level at t_4_) revealed no main effect of Group, *F*(1,26) = .27, *p *= .61, or significant influence of covariates.

### Analysis of valence and arousal data

Repeated measures ANOVA for valence ratings at t_1 _and t_2 _revealed no main effect of Group, *F*(1,31) = .02, *p *= .89, as well as no interaction of Group × Time, *F*(1,31) = .11, *p *= .75. However, there was a main effect of Time, *F*(1,31) = 4.64, *p *= .04, indicating that valence ratings were more positive at t_2 _than t_1 _(compare Table 2). Repeated measures ANOVA for arousal ratings at t_1 _and t_2 _revealed no main effects of Group, *F*(1,31) = .34, *p *= .57, or Time, *F*(1,31) = .35, *p *= .56, or interaction of Group × Time, *F*(1,31) = .06, *p *= .80.

**Table 2:** Valence and arousal ratings

	Stress group		Control group	
	
Time	*LSM*	*SE*	*LSM*	*SE*
Valence				
t_1_	6.82	.48	6.81	.50
t_2_	6.06	.48	6.25	.50
t_3_	5.97	.55	6.72	.57
t_4_	5.74	.55	6.22	.57
Arousal				
t_1_	5.29	.59	5.81	.61
t_2_	5.12	.59	5.38	.61
t_3_	5.60	.52	5.06	.53
t_4_	5.30	.52	5.18	.53

Least square means (LSM) and standard errors (SE) of valence and arousal ratings for each group. The SAM scale ranged from 1 to 9 with 1 = "highly pleasant/low arousing" and 9 = "highly unpleasant/highly arousing".

For valence ratings at t_3 _and t_4_, no main effect of Time, *F*(1,31) = .99, *p *= .33, no difference between groups, *F*(1,30) = .75, *p *= .39, or interaction of Time × Group, *F*(1,31) = .13, *p *= .72, was observed. The covariate (rating at t_2_) was significant, *F*(1,30) = 4.27, *p *= .05, indicating that high ratings at t_2 _also led to higher ratings at t_3 _and t_4_. Similarly, for arousal ratings no difference between groups, *F*(1,30) = .27, *p *= .61, no main effect of Time, *F*(1,31) = .05, *p *= .82, or interaction of Time × Group, *F*(1,31) = .32, *p *= .58, was observed, while the covariate (rating at t_2_) was again significant, *F*(1,30) = 16.05, *p *< .0001, indicating that high ratings at t_2 _also led to higher ratings at t_3 _and t_4_.

### Analysis of visual analog symptom rating scales

Repeated measurement ANOVAs revealed no significant interaction of Time × Group at t_2 _and t_3 _for the ratings of the following symptoms: nightmares, *F*(1,31) = 2.85, *p *= .10, intrusions, *F*(1,31) = .01, *p *= .91, irritability, *F*(1,31) = .62, *p *= .44, sleep disturbances, *F*(1,31) = .01, *p *= .93, and thinking of one's worst event at this very moment, *F*(1,31) = .01, *p *= .91. In contrast, a significant interaction of Time × Group, *F*(1,31) = 4.44, *p *= .04, revealed that the control group exhibited a tendency to rate memories of their worst event as less unpleasant and stressful at t_3 _compared to t_2_, *F*(1,15) = 3.64, *p *= .08, whereas the stressed experimental group showed no such tendency, *F*(1,16) = 1.04, *p *= .32.

With respect to the question of whether memories of the most traumatic event were more intense than before, less intense, or unchanged, no significant differences between the stress and the control group were observed, χ^2^(df = 2) = 1.13, *p *= .57.

### Analysis of the delayed match-to-sample task

Participants performed extremely poorly in the delayed match-to-sample task: they made mistakes in 30.7% of trials at t_2 _and in 36.1% of trials at t_3_. (Note: in a healthy population (9 male, 7 female; mean age 30 years) error rates of 1.5% were observed). No significant effects or interactions on performance were found; notably, participants in the stress group did not perform worse on the second run than the control group, interaction of Time × Group, *F*(1,28.7) = .16, *p *= .70.

The analysis of response latencies showed that participants responded overall faster in the second run of the DMS task compared to the first run, *F*(1,26.3) = 10.66, *p *= .003. However, no main effect of Group, *F*(1,27.3) = 1.42, *p *= .24, or interaction of Time × Group, *F*(1,26.3) = .07, *p *= .80, was observed.

## Discussion

This study found no difference in cortisol excretion in participants in the supposed stress condition as compared to individuals in the control group, although both groups did show the typical diurnal decline in cortisol. Correspondingly, ratings of subjective well-being and PTSD symptoms did not show any significant difference between both groups. The results suggest that a comprehensive diagnostic interview, including questions about traumatic events, is not a stressor that triggers an HPA-axis based alarm response, even for severely traumatized persons such as survivors of torture.

### Cortisol response to a trauma-related stressor

Previous studies commonly used a general stressor (psychosocial, cognitive etc.) and compared cortisol reagibility of traumatized persons with (and without) PTSD to that of a control group being exposed to the same stressor [26,28-30]. In contrast, the present study compared the effect of a stressful versus a non-stressful interview in PTSD patients.

To our knowledge, the only study with a somewhat similar design was conducted by Elzinga et al. [27]. They compared the cortisol responses to personalized trauma scripts of persons with current PTSD to those of persons with life-time PTSD and found increased cortisol secretion during script exposure in the current PTSD compared to the life-time PTSD group. Whereas in the present study subjects were interviewed about their traumatic events with a standardized questionnaire on torture/war experiences, persons in the Elzinga et al. study were read two trauma scripts of approximately 1 minute length describing a severe childhood sexual or physical abuse event (script 1) and a situation in which the person felt alone and abandoned (script 2). Perhaps listening to one's own traumatic experience read aloud in present-tense is a more severe stressor than answering questions about whether one has encountered certain experiences, and thus leads to increased cortisol secretion. However, it must be noted that Elzinga et al. compared the reaction to trauma scripts between patients with lifetime versus patients with current PTSD, while the present study compares the effects of trauma-related questioning with a trauma-irrelevant questionnaire in current PTSD patients; thus the similarity between the Elzinga et al. and the current study is limited.

Finally, the case study of Otte et al. [31] is most directly comparable to the present experimental procedure in that a PTSD patient underwent imaginal exposure therapy, while subjective distress as well as cortisol was measured before, during and after exposure in the first and 20th treatment session. In contrast to our results, subjective distress before and in the first 15 to 30 minutes of the first treatment session was very high, while afterwards subjective distress levels declined. In the present study, participants were already considerably distressed when we started the interview, which may have induced a ceiling effect. However, in agreement with our findings, Otte et al. also observed no accompanying cortisol response in response to imaginal trauma exposure, and cortisol dynamics were essentially the same during the first and the 20th session.

### Possible influences on HPA activity following a stressor

#### Nature of the stressor

A meta-analysis evaluating the influence of chronic stress on the HPA axis found that chronic stress which threatens physical integrity (e.g. combat, traumatic stress) leads to a diurnal profile of cortisol excretion that is high and flat, whereas stress posing a threat to the social self (e.g. divorce), was associated with higher cortisol at specific times in the day, including morning and afternoon/evening [44]. Furthermore, in a meta-analysis evaluating the influence of *acute *stressors on cortisol responses in healthy controls, significant cortisol responses were induced by cognitive tasks (*d *= .2), public speaking/verbal interaction tasks (*d *= .39), and public speaking/cognitive task combinations (*d *= .87). However, the influence of emotion induction or noise exposure were not significant [10].

We currently do not know of a study that compared the influence of various stressors (e.g., psychosocial, cognitive, physical, trauma reminder) in the same patient group. Perhaps some of the inconsistent literature could be attributed to the fact that cortisol responses in traumatized persons vary with the type of stressor applied. A threat to the social self, for instance, would be a likely candidate.

#### Core emotions elicited by the stressor

It has been hypothesized that the direction and magnitude of the HPA response to a stressor is influenced by the emotion(s) elicited by the situation [44]. Consistent with this notion, a recent meta-analysis found that situations likely to elicit shame (e.g. sexual abuse) were associated with significantly higher afternoon/evening cortisol, whereas those evoking loss (e.g. death of spouse) were accompanied by a flattened diurnal profile with lower morning cortisol but higher afternoon/evening cortisol [44]. It is possible that the main emotion(s) elicited through a trauma reminder (e.g., helplessness, fear, sadness, anger/aggression) might also influence the pattern of cortisol excretion. Support for this notion comes from the observation that one patient in the present study became severely aggressive during the diagnostic interview and correspondingly exhibited significantly increased cortisol levels that showed up as extreme outliers and thus had to be excluded from the analysis. To our knowledge, no study so far investigated predominant emotions in PTSD and cortisol profiles and reagibility.

#### Controllability of the stressor

Another influencing factor may be controllability of the stressor. In a meta-analysis, chronic stress that was rated as potentially controllable was associated with significantly higher morning cortisol whereas uncontrollable stress was associated with lower morning cortisol [44]. Similarly, another meta-analysis evaluating the effect of acute stressors on cortisol responses in healthy controls showed that uncontrollable situations led to greater cortisol changes than those that were controllable [10]. Currently, to our knowledge, no study exists that investigated cortisol responses in relation to trauma reminders that also evaluated whether the participating persons experienced the situation as controllable or uncontrollable.

#### Timing of traumatic stressor (early life vs. adulthood)

Exposure to chronic or traumatic stress early in life, when the nervous system is still developing, may result in distinct alterations of the HPA axis compared to when the stressor occurs later in life. Support for this notion comes from a study by Heim et al. [30] who found a persistent sensitization of the HPA axis and autonomic stress response in women with severe early life stress. However, Santa Ana et al. [28] did not find differences between child or adult trauma with respect to ACTH secretion – both groups showed a flattened ACTH response.

## Conclusion

A comprehensive diagnostic interview, including extensive questioning of traumatic events does not seem to cause an HPA-axis based alarm response or changes in psychological measures, even for severely traumatized persons such as survivors of torture. Nevertheless, the present results should not be taken to mean that questioning about traumatic experiences is not taxing for both patients with PTSD and interviewers. On the contrary, interviews should continue to be conducted within a secure, supportive and empathic environment in which the interviewee has as much control over the situation as possible. In conclusion, assuming that basic therapeutic variables are met, we do not have any psychophysiological evidence that a careful trauma diagnosis floods a trauma survivor with stress hormones or causes them additional distress.

## Competing interests

The author(s) declare that they have no competing interests.

## Authors' contributions

ITK designed the study, performed part of the data acquisition, analyzed the data, and wrote the manuscript. CE designed the study and acquired data together with ITK. MR supported data acquisition. FN supported data acquisition and supervised interviewers. DJFD designed the study and supported manuscript writing. TE designed the study and supported each stage of the experiment and manuscript writing. All authors read and approved the final manuscript.

## Pre-publication history

The pre-publication history for this paper can be accessed here:


